# From research to practice: Implementing an experimental home treatment model into routine mental health care

**DOI:** 10.1192/j.eurpsy.2020.91

**Published:** 2020-10-13

**Authors:** N. Stulz, W. Kawohl, M. Jäger, S. Mötteli, U. Schnyder, U. Hepp

**Affiliations:** 1 Integrated Psychiatric Services Winterthur—Zurcher Unterland, P.O. Box 144, CH-8408 Winterthur, Switzerland; 2 Psychiatric Services Aargau, P.O. Box 432, CH-5201 Brugg, Switzerland; 3 KPPP, University Hospital of Psychiatry Zurich, Lenggstrasse 31, CH-8008 Zurich, Switzerland; 4 Psychiatrie Baselland, Bienentalstrasse 7, CH-4410 Liestal, Switzerland; 5 University of Zurich, CH-8001 Zurich, Switzerland

**Keywords:** acute care, external validity, mobile teams, outreach services

## Abstract

**Background:**

In line with previous findings, in a recent randomized controlled trial (RCT), we found that home treatment (HT) for acute mental health care can reduce (substitute) hospital use among severely ill patients in crises. This study examined whether the findings of the RCT generalize to HT services provided under routine care conditions.

**Methods:**

We compared patients who received HT during the RCT study phase with patients who received the same HT service after it had become part of routine mental health services in the same catchment area. Sociodemographic and clinical characteristics as well as service use (HT and hospital bed days) were compared between the RCT and the subsequent routine care study period.

**Results:**

Compared to patients who received HT during the RCT, routine care HT patients were more often living with others, less often admitted compulsorily, more often diagnosed with anxiety and stress-related disorders (ICD-10 F4) and less often diagnosed with schizophrenia spectrum disorders (F2). When compared to patients who were exclusively treated on hospital wards, involvement of the HT team in patients’ care was associated with a clear-cut reduction of hospital bed days both during the RCT and under routine care conditions. However, unlike during the RCT study period, involvement of HT was associated with longer overall treatment episodes (inpatient + HT days) under routine care conditions.

**Conclusions:**

HT seems to reduce the use of hospital bed days even under routine care conditions but is at risk of producing longer overall acute treatment episodes.

## Introduction

Home treatment (HT) has been proposed as an alternative to acute care on hospital wards for mentally ill patients [1–3]. Mobile and multi-professional HT teams are available around the clock to provide intensive care in the patient’s domestic environment, whenever feasible. Such teams may visit patients several times daily. However, their interventions are restricted to acute crises and should not exceed the length of an otherwise indicated hospital stay (typically days or weeks). From a clinical perspective, HT is intended to be more acceptable to certain service users than hospital admission and may provide better opportunities to address social factors potentially contributing to many crises [1]. Crisis resolution and HT teams have been widely implemented in various mental health systems worldwide [4–9]. Evidence to support their effectiveness has remained moderate, however [10]. In a recent randomized controlled trial (RCT), we found that HT can reduce (substitute) hospital use among severely ill patients in crises and seems to result in comparable clinical/social outcomes and patient satisfaction as standard inpatient care [11]. This is largely in line with the findings of previous RCTs on crisis resolution and home treatment (HT) teams [10,12]. However, RCTs have sometimes been criticized for limited external validity [13]. Factors such as rigorous inclusion/exclusion criteria or (unblinded) clinical staff who is particularly motivated when examining new interventions might limit the generalization of findings derived from RCTs to routine clinical care [14]. Even though a recent Cochrane review found comparable results for healthcare interventions in RCTs and in observational studies [15], complex mental health system interventions, in particular, might not work equally well in RCTs as compared to natural routine care settings [16,17].

In this follow-up study, we aimed at examining whether patients who received HT for acute care during the study period of the RCT differed from patients who received the same HT service after the HT model had become part of routine mental health services in our catchment area. In order to do so, we compared HT patients between the two implementation phases (RCT vs. routine care) in terms of sociodemographic and clinical characteristics as well as regarding the use of “inpatient-equivalent” HT services.

## Methods

### Setting

Data were collected in the Federal State of Aargau (Switzerland). Psychiatric Services Aargau AG (PDAG) is legally bound to provide mental health care to the Aargau population (approximately 680,000 inhabitants) in one psychiatric hospital (128 beds on acute wards for adult general psychiatry) and several day hospitals and outpatient clinics. A central triage unit with a highly experienced staff is responsible for gatekeeping and ensures that only patients who require immediate inpatient treatment are hospitalized (day or outpatient treatment is given preference whenever feasible) [18]. For more details, see [11].

In 2015, a mobile and multi-professional HT team was established at the PDAG’s psychiatric hospital to provide acute outreach mental health care to the population in the service area 24 hours a day and 7 days a week [11]. Organisationally, the HT team was closely linked to the central triage unit and could provide intensive acute care at home once an experienced triage unit clinician had deemed inpatient treatment necessary. This procedure was in use for the whole study period examined here in order to ensure that only patients who unequivocally required inpatient treatment were considered for “inpatient-equivalent” HT.

### Sample and procedures

#### Study phase 1: RCT

During a 1-year enrolment period (April 14, 2015 to April 13, 2016), we randomized all patients for whom immediate inpatient treatment was deemed necessary by the central triage unit [18] to either a service model including the HT alternative to hospital care (experimental group) or to a conventional service model that provided standard inpatient care only (control group). All randomized patients had to meet the following inclusion criteria: (a) 18 to 65 years old; (b) permanent private address (no residential accommodation), reachable by car within 30 minutes from the HT base at the psychiatric hospital (this criterion applied to approximately 80% of all inhabitants in the service region); (c) one of the following primary diagnoses according to ICD-10 [19]: F2, F3, F4, F5, F6, F8, F9, or Z; (d) being referred to the department of general psychiatry (i.e., referrals to specialized wards, such as forensics were excluded); (e) basic health insurance (patients with supplementary “private” health insurance plans were treated on different hospital wards); and (f) sufficient German language skills to communicate without a translator. In addition, patients with alcohol, cocaine, or opioid dependence and patients with mental retardation or organic mental disorders were excluded, regardless of whether one of the listed disorders was the primary or a secondary diagnosis [11]. Importantly, initiation of HT was of course not mandatory for patients in the experimental group of the RCT; it merely provided a possible alternative to acute inpatient care depending on clinical considerations and preferences of patients and relatives [11].

#### Study phase 2: routine care

All of the aforementioned inclusion criteria for HT were maintained during the first year after randomization had been stopped, that is, when HT became part of the routine services in our catchment area. Between April 14, 2016 and April 13, 2017, all patients in crises who met these inclusion criteria and who were deemed in need of inpatient treatment by the triage unit could be considered for HT instead of inpatient treatment by clinical staff. Because the number of HT slots remained stable after the termination of the RCT but randomization to the control group was stopped, the number of potential HT candidates was increased in routine care (see below).

To sum up briefly, staff of the hospital wards and the HT team could suggest HT as an alternative to inpatient care to all patients who (a) met the inclusion criteria (identical in both study phases); (b) were deemed in need of immediate inpatient treatment by the triage unit (both study phases); and (c) had been randomized to the experimental arm with the new services model including the HT option (RCT phase only). HT was initiated only if it appeared clinically appropriate and safe, and if patients and their relatives agreed.

### Interventions

The structures and processes of HT services did not differ between the RCT phase and the routine care phase of this study. A multi-professional team aimed to manage crises at the patients’ homes in both study phases if feasible. The central triage unit and hospital wards could refer patients to “inpatient-equivalent” HT services at any time during acute treatment episodes once inpatient treatment had been deemed necessary by an experienced central triage unit clinician. Staff at the central triage unit and on hospital wards was instructed to offer HT to patients at admission or any time during the acute inpatient treatment episode as soon as it appeared clinically appropriate and safe. If patients or their relatives refused HT or if patients were not offered HT (e.g., due to clinical considerations such as acute suicidality), they were treated exclusively on hospital wards. The 12 HT slots were operated by a senior psychiatrist (0.9 full-time equivalents (FTE)), two clinical psychologists (1.6 FTE), nurses (6.5 FTE), a social worker (0.6 FTE), and a team assistant (1.0 FTE). Staff was generally available 24 hours (on call from 10:00 pm to 8:00 am). Patients were typically visited at home once daily for approximately 1 h, with the option for multiple visits a day (or night) if necessary. Interventions were individually tailored but included typical ingredients of acute care, such as crisis intervention, pharmacotherapy, psychoeducation, brief psychotherapy, and intervention by a social worker. HT patients could take part in therapeutic group sessions in our mental hospital but they had to organize transportation themselves. Unfortunately, we were not able to offer transportation services for HT patients due to financial restrictions.

### Data collection

Information on patients’ sociodemographics, routine clinical diagnoses, and service use were drawn from clinical records and from the case register in the medical database of the PDAG.

### Data analysis

Sociodemographic and clinical characteristics of the HT patients were compared between the two study phases (RCT vs. routine care) using *χ* ^2^, or independent-samples *t*-tests. Data on service utilization (i.e., the number of HT days and hospital bed days per patient) were compared between patient groups using *t*-tests since they had been shown to outperform Wilcoxon–Mann–Whitney tests in large samples even if data is highly skewed [20]. If there were multiple admissions (treatment cases) per patient during the 12-month enrolment period of the RCT or the 12-month routine care phase, respectively, analyses were restricted to the first (index) treatment episode within these periods. Statistical analyses were performed using SPSS version 24.

The responsible regional ethics committee declared that this research project does not fall under the remit of the cantonal or federal law (Human Research Act) because it evaluated anonymized data only. However, the ethics committee has reviewed the submitted documents and confirmed that the research project fulfills the general ethical and scientific standards for research with humans (EKNZ Req. 2020–00528).

## Results

### Patients

**Figure 1.**
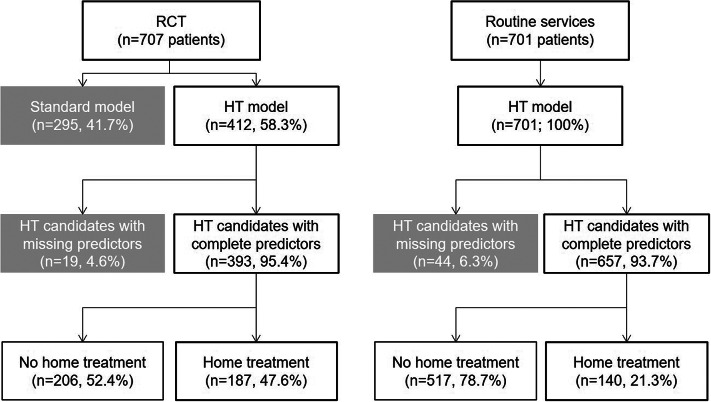
Patient selection. Note: HT = home treatment.

Figure 1 shows the selection of the patients for this study. During the 1-year enrolment period of the RCT, 412 (58.9%) of the 707 eligible patients with an immediate need for hospital admission had been randomized to the new service model with the option of providing HT instead of inpatient care. The remaining patients (*n* = 295) were randomized to a standard care model without the option of HT (control group). These controls were not further considered in the current analyses.

During the first year after stopping the enrolment for the RCT, 701 patients were referred to the psychiatric hospital, were deemed in need of hospital admission by the central triage unit and met the inclusion criteria for HT (which had remained unchanged as compared to the RCT). All 701 patients could be referred to the HT services by the clinical staff of the triage unit or the hospital wards. After excluding patients with missing data on sociodemographic or clinical characteristics, there were 393 (95.4%) HT candidates with full information during the RCT phase and 657 (93.7%) HT candidates during the first year after the RCT (Figure 1).

### Comparison of HT patients during the RCT and in routine care

Home treatment patients in routine care were more often living with others than HT patients enrolled in the RCT, and they had less often been admitted compulsorily to acute care (Table 1). Neurotic, anxiety, and stress-related disorders (ICD-10 F4) were more prevalent in routine care than HT patients, whereas schizophrenia, schizotypal, and delusional disorders (F2) were more prevalent in the HT patients of the RCT (Table 1).

**Table 1.** Characteristics of home treatment patients.

Patient characteristics	RCT	Routine care	*p*
	(*n* = 187)	(*n* = 140)	
Age (years), *M* (*SD*)^a^	40.5 (11.8)	41.5 (11.8)	0.445
Sex: female (vs. male), *n* (%)^b^	112 (59.9%)	94 (67.1%)	0.179
Citizenship: Foreign (vs. Swiss)^b^	34 (18.2%)	19 (13.6%)	0.263
Living with others (vs. living alone)^b^	122 (65.2%)	111 (79.3%)	0.005
Job status: yes (vs. no)^b^	119 (63.6%)	87 (62.1%)	0.782
Compulsory admission: yes (vs. no)^b^	42 (22.5%)	14 (10.0%)	0.003
Primary diagnosis (ICD-10)^b^ F2 (Schizophrenia or schizoaffective disorder) F3 (Affective disorder) F4 (Anxiety, stress-related, or somatoform disorder) Other	53 (28.3%) 104 (55.6%) 25 (13.4%) 5 (2.7%)	21 (15.0%) 76 (54.3%) 33 (23.6%) 10 (7.1%)	0.002

*Note:* ICD-10: International Classification of Diseases, 10th revision.

a Independent samples *t*-test.

b *χ* ^2^-test.

### Utilization of acute inpatient and HT services

The HT team (offering 12 treatment slots) was involved in the treatment of 187 patients during the one-year enrolment period of the RCT and in the treatment of 140 patients during the first year thereafter, when the HT model had become part of routine services (Figure 1). The vast majority of these HT patients also used some hospital bed days; particularly during the RCT (97.9%) but also after HT had become part of routine services (84.3%; *p* < 0.001). In both study phases, hospital bed days were typically provided immediately after admission to acute care, before patients were subsequently referred to HT services once they had gained sufficient stabilization on the hospital ward.

**Figure 2.**
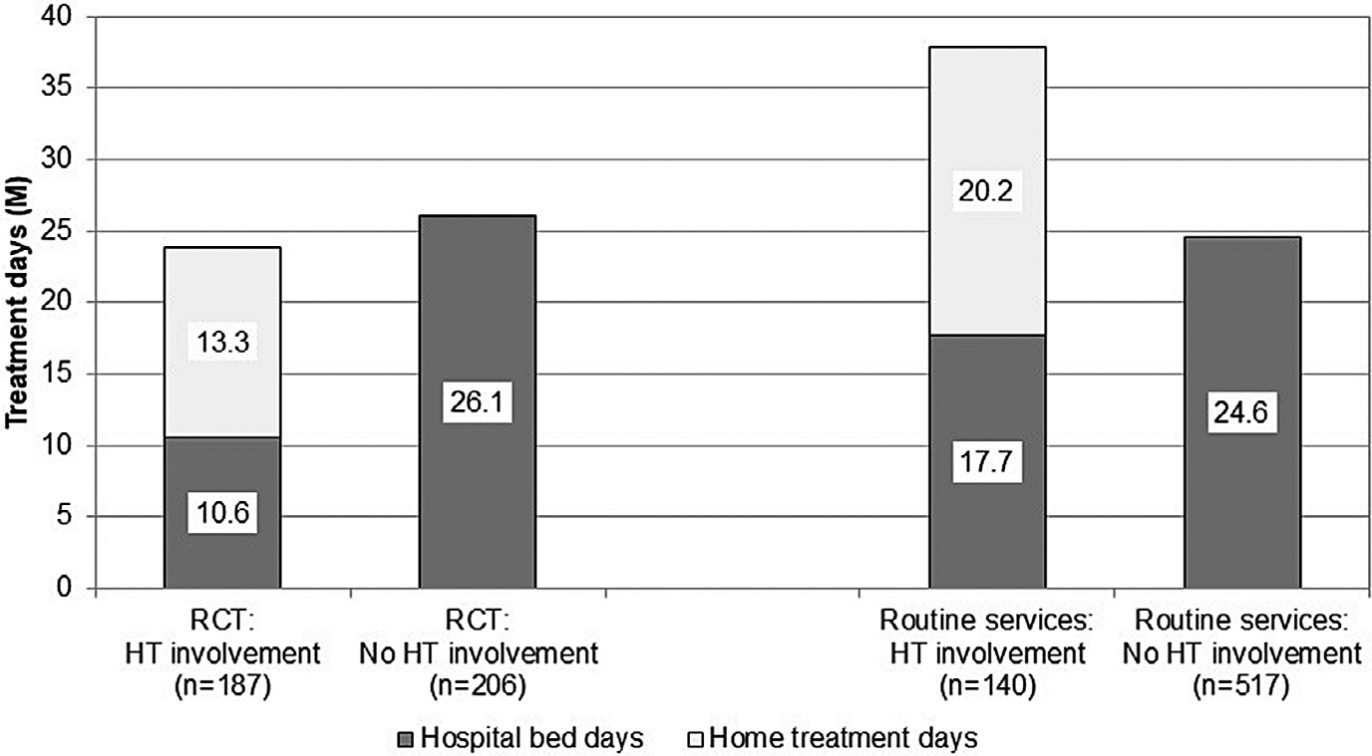
Treatment days per patient. Note: HT = home treatment.

Figure 2 shows the use of acute services (hospital bed days and HT days) during the RCT and the routine care phase. When compared to treatment episodes without HT, involvement of the HT team in patients’ care was associated with a clear-cut reduction of hospital bed days both during the RCT (*M*(SD) = 10.6 (11.9) vs.26.1 (28.6) days, *t*(391) = 6.922, *p* < 0.001) and after HT had become part of routine services in our catchment area (*M*(SD) = 17.7 (20.0) vs. 24.6 (28.5) days, *t*(655) = 2.689, *p* = 0.007). During the RCT, the overall length of acute treatment (inpatient + HT days) did not statistically significantly differ between patients with and without HT (*M*(SD) = 23.9 (13.7) vs. 26.1 (28.6) days, *t*(391) = 0.970, *p* = 0.333). However, after HT had become part of routine mental health services, involvement of the HT team in patients’ care was associated with longer overall treatment episodes when compared to patients who had been exclusively treated on hospital wards (*M*(SD) = 37.9 (23.3) vs. 24.6 (28.5) days, *t*(655) = 5.068, *p* < 0.001). In line with this, HT patients in routine care had longer acute treatments than had HT patients during the RCT both on hospital wards (*M*(SD) = 17.7 (20.0) vs. 10.6 (11.9) days, *t*(325) = −4.027, *p* < 0.001) and in HT (*M*(SD) = 20.2 (9.9) vs. 13.3 (6.8) days, *t*(325) = −7.379, *p* < 0.001).

## Discussion

When being part of routine psychiatric services, involvement of the HT team in patients’ acute care was associated with a clear-cut reduction of hospital bed days (when compared to treatment episodes without HT involvement). However, the extent to which HT reduced the use of hospital bed days was smaller than in the preceding RCT evaluating the same HT service [11]. In addition, involvement of the HT team in patients’ care resulted in longer overall treatment episodes (HT + hospital bed days) in the routine care setting. The overall length of acute care episodes with involvement of the HT team in our routine care setting was very similar to the mean length of treatment episodes in another Swiss HT service [8,21]. In addition, the median number of hospital bed days among HT patients was rather similar in our study (Md = 10.5 days) and in a German HT model (Md = 12 days) under routine conditions [22]. From a resource consumption perspective, HT seems to reduce the use of hospital bed days even under routine care conditions but is at risk of resulting in longer overall acute treatment episodes.

### Limitations

Several limitations of this study have to be addressed. Firstly, this was an observational study. We do not know whether the association between involvement of an HT team and reduction (substitution) of hospital bed days differed between the two study phases only because of the discontinuation of randomization or whether it was also influenced by other factors. Secondly, information on sociodemographic and clinical characteristics of patients was very limited and fully relied on routinely collected data. The validity of routine clinical diagnoses has been demonstrated in a stratified subsample (*n* = 100) of the RCT, though [23]. Thirdly, even though the Health of the Nations Outcome Scales (HoNOS) [24] are mandatorily administered to all patients in Swiss psychiatric hospitals, we deliberately refrained from comparing HoNOS scores between HT patients in our RCT and HT patients in our routine care phase. The HoNOS had become relevant for a new remuneration system for inpatient care in Switzerland during the ongoing study. This was accompanied by a systematic increase in HoNOS scores at intake both in our hospital and in Swiss psychiatric hospitals in general [25]. Comparisons of HT scores between the two study phases would therefore have been flawed. Fourth, we had no clinical and social outcome parameters in our study. Instead, we focused on service use (HT and hospital bed days) which can be considered a “hard” and crucial outcome indicator with regard to mental health services planning and organization. Finally, our analyses were restricted to the first treatment episode of each patient after his or her enrolment into this study. This precludes any inferences about the long-term impact of HT under RCT versus routine care conditions.

### Possible explanations why service use of HT patients differed under RCT and routine care conditions

When designing our RCT we aimed to emphasize its external validity by using measures such as relatively broad inclusion criteria and a Zelen design [26] which enabled recruitment of patients in crises who were unable to make informed decisions at admission [11]. The single randomized consent design suggested by Zelen proposes randomization of all patients without their previous agreement and irrespective of their clinical condition. Written informed consent is subsequently only obtained from patients in the experimental group (here patients who were actually offered HT) [27]. Despite these measures to strengthen the external validity, the results of our RCT were found to generalize only partially to a routine care setting. There are several possible explanations why the same HT team had a different impact on service use when acting in an RCT or in a routine care setting in the same catchment area.

Firstly, the patients who received HT differed between the two study phases in some characteristics. When compared to the RCT, HT patients in a routine care setting were less often diagnosed with schizophrenia (F2) and less often involuntarily admitted to acute treatment. On the other hand, they were more often diagnosed with anxiety and stress-related disorders (F4) and more often co-habiting with others. These findings suggest a shift of the focus of the HT team between the two study phases—away from HT patients with severe mental disorders in highly acute states toward more prolonged treatments for patients with better social functioning in routine care. The number of potential HT candidates had massively increased (by >50%) after HT had become part of routine services (because no more patients were randomized to a control condition without HT option). Therefore, during the RCT, there might have been more pressure for the HT team to include even severely ill patients in order to use the 12 HT slots to capacity and to meet the RCT’s inclusion target. This was also suggested by anecdotal reports of some HT team members.

Secondly, and in line with the aforementioned, the pressure to provide efficient (short) treatments might have generally decreased to some degree after the termination of the RCT. It is well-known that individuals tend to behave differently when they are aware of being under observation in a study (Hawthorne effect [28]). The effects of experimental interventions in RCTs might be overestimated if unblinded clinicians are particularly enthusiastic and motivated because they are belonging to the “avant-garde” who is applying innovative interventions for the first time.

Last but not least, coincidentally with the termination of the RCT, the position of the head of department became vacant for most of the time of the post-randomization study period. Experiences in routine mental health care show that treatments may tend to get longer and longer if there are no incentives to keep them as long as necessary but as short as possible. While this might be true in any treatment setting, in a new outreach treatment alternative to substitute acute inpatient care, it seems to be particularly important to repeatedly remind the HT staff to treat patients only as long as they would be otherwise treated in acute hospital wards. We cannot exclude that the lack of guidance from the top level also contributed to extended lengths of HT in our routine care setting.

## Conclusions

Involvement of an HT team in patients’ acute care seems to reduce the use of hospital bed days in a routine care setting. However, when compared to an RCT setting, the extent to which HT reduces the use of hospital bed days may be smaller and overall acute treatment episodes (including both HT and hospital bed days) may be longer.

## Data Availability

Data cannot be shared due to very strict data protection laws in Switzerland.
